# Hyperactive S6K1 Mediates Oxidative Stress and Endothelial Dysfunction in Aging: Inhibition by Resveratrol

**DOI:** 10.1371/journal.pone.0019237

**Published:** 2011-04-22

**Authors:** Angana G. Rajapakse, Gautham Yepuri, João M. Carvas, Sokrates Stein, Christian M. Matter, Isabelle Scerri, Jean Ruffieux, Jean-Pierre Montani, Xiu-Fen Ming, Zhihong Yang

**Affiliations:** 1 Department of Medicine, Division of Physiology, University of Fribourg, Fribourg, Switzerland; 2 Cardiovascular Research, Institute of Physiology, University of Zurich and Cardiology, University Hospital Zurich, Zurich, Switzerland; University of Padova, Medical School, Italy

## Abstract

Mammalian target of rapamycin (mTOR)/S6K1 signalling emerges as a critical regulator of aging. Yet, a role of mTOR/S6K1 in aging-associated vascular endothelial dysfunction remains unknown. In this study, we investigated the role of S6K1 in aging-associated endothelial dysfunction and effects of the polyphenol resveratrol on S6K1 in aging endothelial cells. We show here that senescent endothelial cells displayed higher S6K1 activity, increased superoxide production and decreased bioactive nitric oxide (NO) levels than young endothelial cells, which is contributed by eNOS uncoupling. Silencing S6K1 in senescent cells reduced superoxide generation and enhanced NO production. Conversely, over-expression of a constitutively active S6K1 mutant in young endothelial cells mimicked endothelial dysfunction of the senescent cells through eNOS uncoupling and induced premature cellular senescence. Like the mTOR/S6K1 inhibitor rapamycin, resveratrol inhibited S6K1 signalling, resulting in decreased superoxide generation and enhanced NO levels in the senescent cells. Consistent with the data from cultured cells, an enhanced S6K1 activity, increased superoxide generation, and decreased bioactive NO levels associated with eNOS uncoupling were also detected in aortas of old WKY rats (aged 20–24 months) as compared to the young animals (1–3 months). Treatment of aortas of old rats with resveratrol or rapamycin inhibited S6K1 activity, oxidative stress, and improved endothelial NO production. Our data demonstrate a causal role of the hyperactive S6K1 in eNOS uncoupling leading to endothelial dysfunction and vascular aging. Resveratrol improves endothelial function in aging, at least in part, through inhibition of S6K1. Targeting S6K1 may thus represent a novel therapeutic approach for aging-associated vascular disease.

## Introduction

Aging is a dominant risk factor for cardiovascular disease [1]. One of the important features of vascular aging is endothelial dysfunction characterized by decreased vasoprotective endothelial nitric oxide (NO) bioavailability resulting from numerous mechanisms including oxidative stress [2]. Excessive generation of reactive oxygen species (ROS) such as superoxide anion in the aging vasculature inactivates NO [2], [3], leading to endothelial dysfunction in aging [3], [4]. However, the source and mechanisms of enhanced oxidative stress in endothelial aging remain incompletely understood.

Evidence is emerging that mTOR/S6K1 signalling is an important regulator of aging [5] and aging-associated disorders including cardiovascular diseases [6]. Early studies demonstrated that inhibition of mTOR signalling is capable of extending lifespan in invertebrates [7]–[11]. These observations have been recently extended to mice [12]–[14]. Studies also suggest that lifespan extension by dietary restriction in several species including mice is possibly mediated by reduction of mTOR/S6K1 signalling [6]. mTOR is an evolutionarily conserved serine/threonine protein kinase which integrates multiple signalling pathways regulating gene expression involved in metabolism, cell survival, and cell proliferation [15]. With other molecular components, mTOR forms two structurally and functionally distinct complexes namely mTORC1 and mTORC2. mTORC1 regulates cell growth through S6K1 and eIF-4E–binding protein 1 (4E-BP1), is sensitive to the immunosuppressant rapamycin, whereas mTORC2 exerts its effects through Akt and is rapamycin-insensitive [16]. Therefore, most of the inhibitory effects of rapamycin are attributable to the inhibition of signalling mediated by mTORC1-S6K1 [16]. Evidence for a role of S6K1 in regulation of mouse lifespan has been presented recently [13], suggesting that the aging-modulating effect of mTOR is mediated through its downstream effector S6K1. Although there is evidence for a role of mTOR/S6K1 in vascular functions [17], only little information is available about the role of mTOR, particularly of S6K1, in aging-associated vascular dysfunctions.

In particular, experiments analysing cardiovascular mTOR/S6K1 activity in aging animal models yield inconsistent results. A study using microarray analyses showed that gene expression pattern associated with mTOR is suppressed upon aging in the heart of Fischer 344 rats [18], suggesting that mTOR pathway is down-regulated in aging. Another study, however, showed an increased basal mTOR-mediated phosphorylation of S6K1 at Thr389 in aortas of Fischer 344 x Brown Norway F1 hybrid rats [19], which implicates an enhanced mTOR/S6K1 signalling in aging.

It is now well recognized that resveratrol, a natural polyphenol, exerts protective effects on vascular diseases, type II diabetes, and aging in several animal species [20], [21]. Although there is substantial evidence suggesting that the beneficial effects of resveratrol are attributed to activation of the class III histone deacetylase (HDAC) Sirt1 [22], a recent rigorous study analysing the pharmacological effects of resveratrol and Sirt1-activating drugs has challenged this notion [23]. Moreover, Sirt1-independent effects of resveratrol have also been reported [24]–[26]. At the molecular level, it has been shown that resveratrol is able to inhibit mTOR/S6K1 pathway in different cell types [24], [25], [27]–[29]. However, whether resveratrol improves endothelial function in aging through inhibition of S6K1 is not known.

These findings prompted us to investigate whether S6K1 plays a role in endothelial dysfunction in aging and whether resveratrol protects against endothelial dysfunction through inhibition of S6K1 signalling under the aging condition.

## Results

### Enhanced S6K1 activity, increased superoxide and decreased nitric oxide (NO) levels in senescent endothelial cells

**Figure 1:**
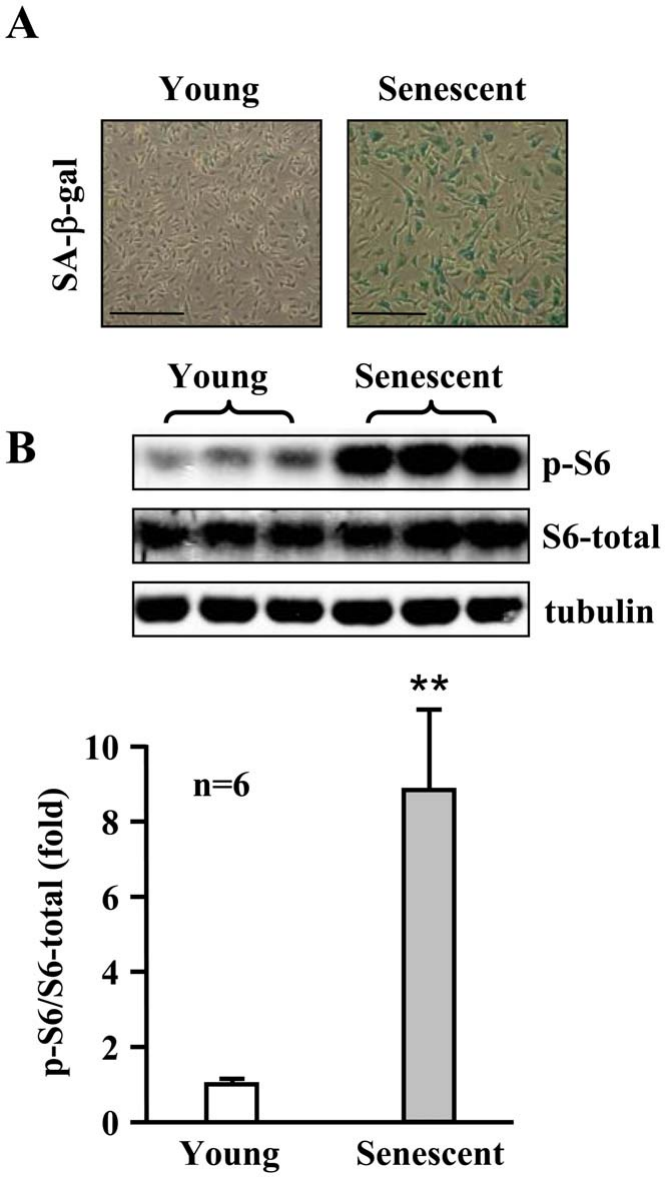
Increased S6K1 activity in senescent endothelial cells. (**A**) A representative SA-ß-gal staining of young and senescent HUVECs. (**B**) Immunoblotting analysis of S6-S235/S236 (p-S6), total S6, and tubulin protein levels in the young and senescent endothelial cells. Quantification of the signals is shown in the lower panel. n = 6 in each group. **p<0.01. Scale bar = 0.2 mm.

**Figure 2:**
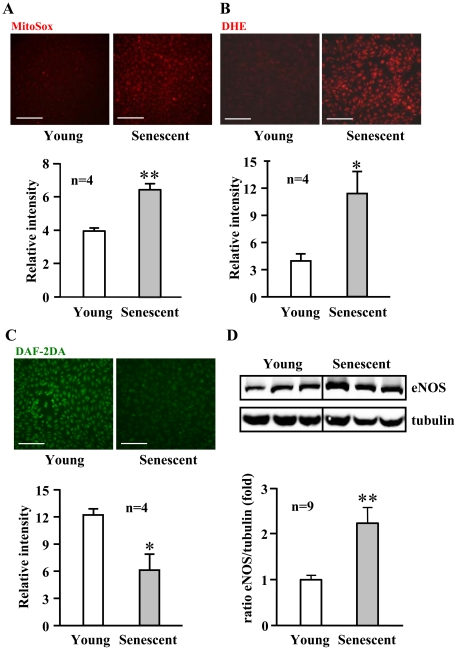
Increased oxidative stress in senescent endothelial cells. Fluorescent signals of (**A**) MitoSox, (**B**) DHE, and (**C**) DAF-2DA staining for detection of mitochondrial, cytoplasmic superoxide generation (red) and NO production (green) in young and senescent HUVECs, respectively. Quantification of the signals from four independent experiments of each group is shown in the corresponding lower panels. *p<0.05, **p<0.01 vs. young cells. Scale bar = 0.2 mm. (**D**) Immunoblotting showing increased eNOS protein level in senescent cells. Quantification of eNOS protein level is shown in the lower panel. n = 9 in each group. **p<0.01 vs. young cells.

To investigate the role of S6K1 in endothelial aging, we first determined S6K1 activity in cultured young and senescent human endothelial cells. The senescence status of the cells was confirmed by higher number of cells which stained positively for senescence-associated ß-galactosidase (SA-ß-gal, **Fig. 1A**). A significantly higher S6K1 activity as measured by phosphorylation of its substrate S6 at serine 235/236 (S6-S235/S236) was detected in senescent cells when compared with young cells (**Fig. 1B**, n = 6, p<0.01). The increased S6K1 activity in the senescent cells was associated with an enhanced superoxide production and a decreased NO level as detected by MitoSox and dihydroethidium (DHE) staining, and by 4,5-diaminofluorescein diacetate (DAF-2DA) staining, respectively (**Fig. 2A to 2C**). The eNOS protein level was, however, significantly higher in senescent cells as compared to young cells (**Fig. 2D**).

### eNOS uncoupling in senescent endothelial cells

**Figure 3:**
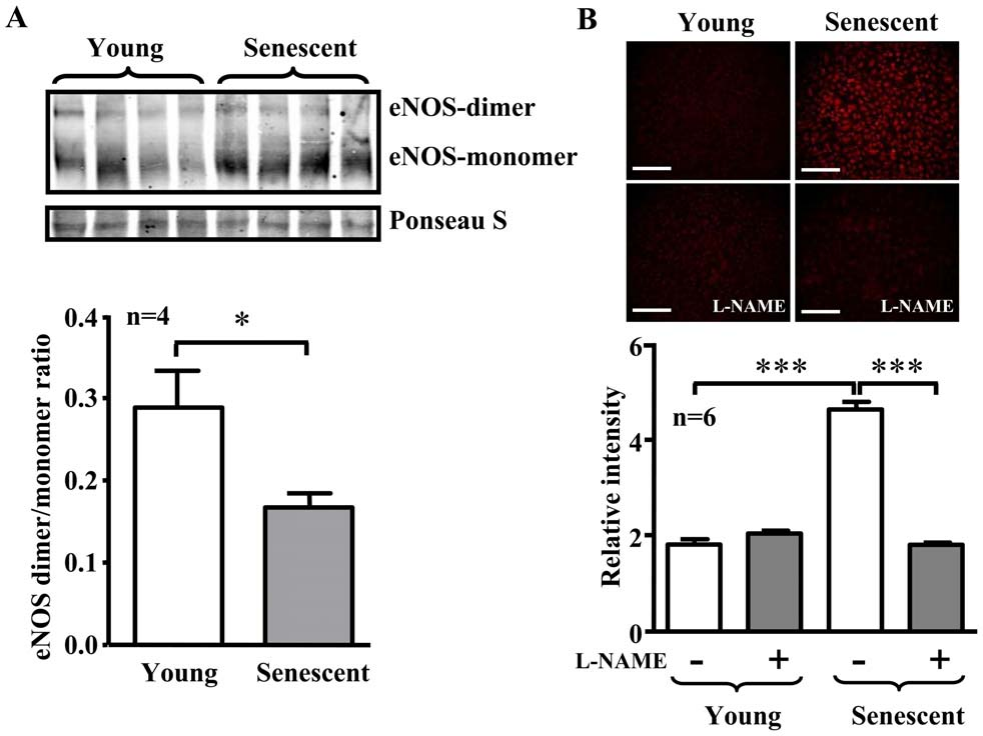
eNOS uncoupling in senescent endothelial cells. (**A**) Immunoblotting analysis of eNOS-dimers and -monomers in the young and senescent HUVEC endothelial cells. Ponseau S staining served as loading control. Quantification of the eNOS-dimer/monomer is shown in the lower panel. n = 4 in each group. *p<0.05. (**B**) DHE staining for detection of cytoplasmic superoxide generation in young and senescent endothelial cells with or without pre-treatment of the cells with L-NAME (1 mmol/L) for 1 hour as indicated. Quantification of the signals from six independent experiments of each group is shown in the corresponding lower panels. ***p<0.001 between indicated groups. Scale bar = 0.2 mm.

To examine whether the increased eNOS protein level with dysfunctional enzymatic activity in senescent endothelial cells is the result of eNOS uncoupling, experiments with low temperature SDS-PAGE for detection of eNOS dimer/monomer were performed. Indeed, eNOS monomer level was significantly higher in the senescent endothelial cells as compared to the young cells, which leads to a significant decrease in eNOS-dimer/monomer ratio in the senescent cells (**Fig. 3A**, n = 4, p<0.05). Moreover, the increased production of superoxide anion in the senescent cells as detected by DHE staining was inhibited by the eNOS inhibitor L-NAME (**Fig. 3B**). These results provide evidence for eNOS uncoupling in senescent endothelial cells.

### Causal role of S6K1 in eNOS uncoupling and endothelial cell senescence

**Figure 4:**
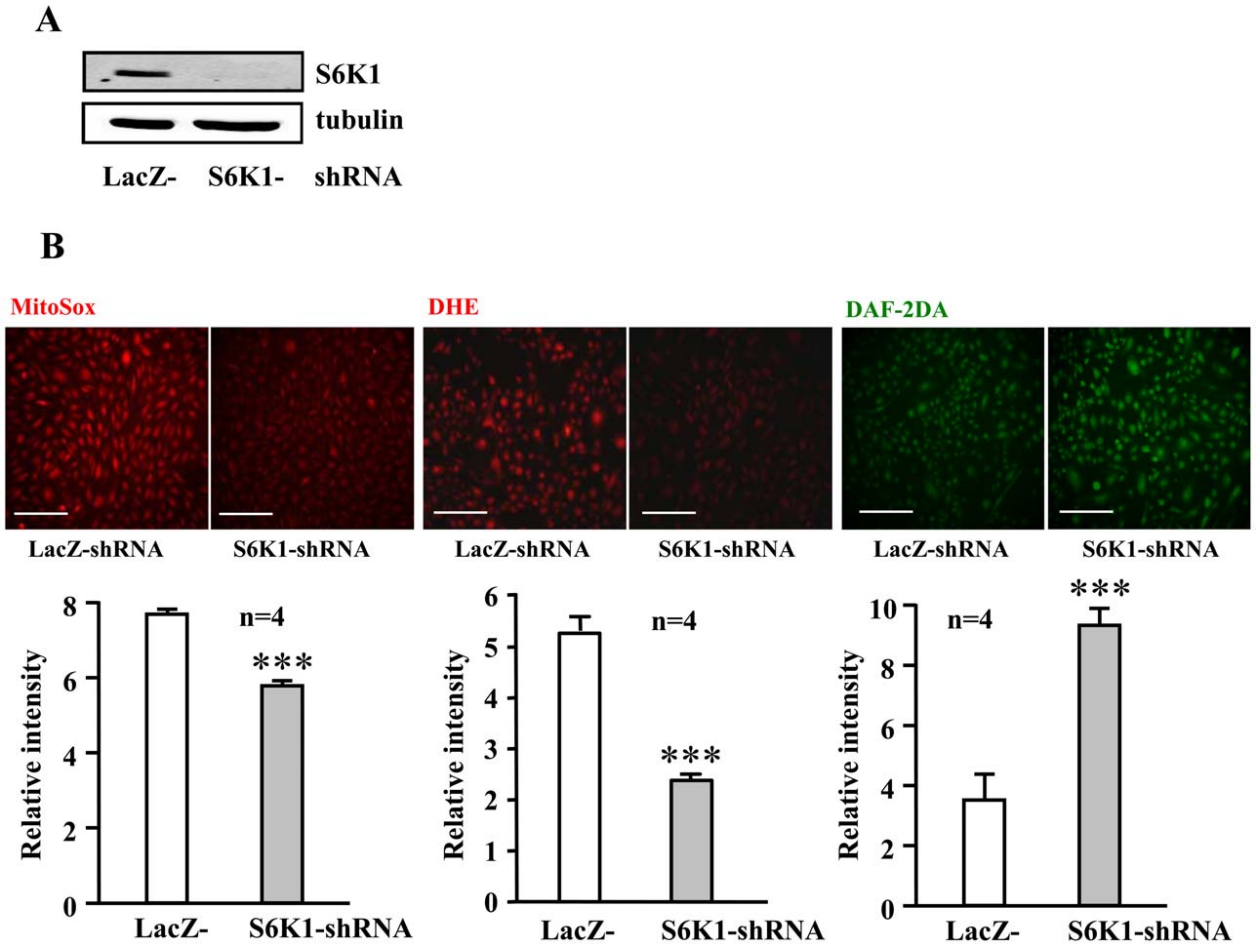
Silencing S6K1 reduces superoxide and enhances NO production in senescent endothelial cells. Senescent HUVECs were transduced either with rAd/U6-LacZ^shRNA^ as control or with rAd/U6-S6K1^shRNA^. Four days post transduction, cells were subjected to (**A**) immunoblotting analysis to reveal the effective silencing of S6K1 and (**B**) MitoSox, DHE, and DAF-2DA staining. Quantification of the signals from four independent experiments of each group is shown in the corresponding lower panels. ***p<0.001 vs. rAd/U6-LacZ^shRNA^ control cells. Scale bar = 0.2 mm.

To test whether the enhanced S6K1 accounts for the increased superoxide and decreased NO production in senescent endothelial cells, S6K1 was knocked down by shRNA-mediated silencing. Silencing S6K1 as demonstrated by decreased protein levels of S6K1 (**Fig. 4A**) significantly reduced oxidative stress as detected by MitoSox and DHE staining, and enhanced NO production as detected by DAF-2DA staining in the senescent endothelial cells (**Fig. 4B**).

**Figure 5:**
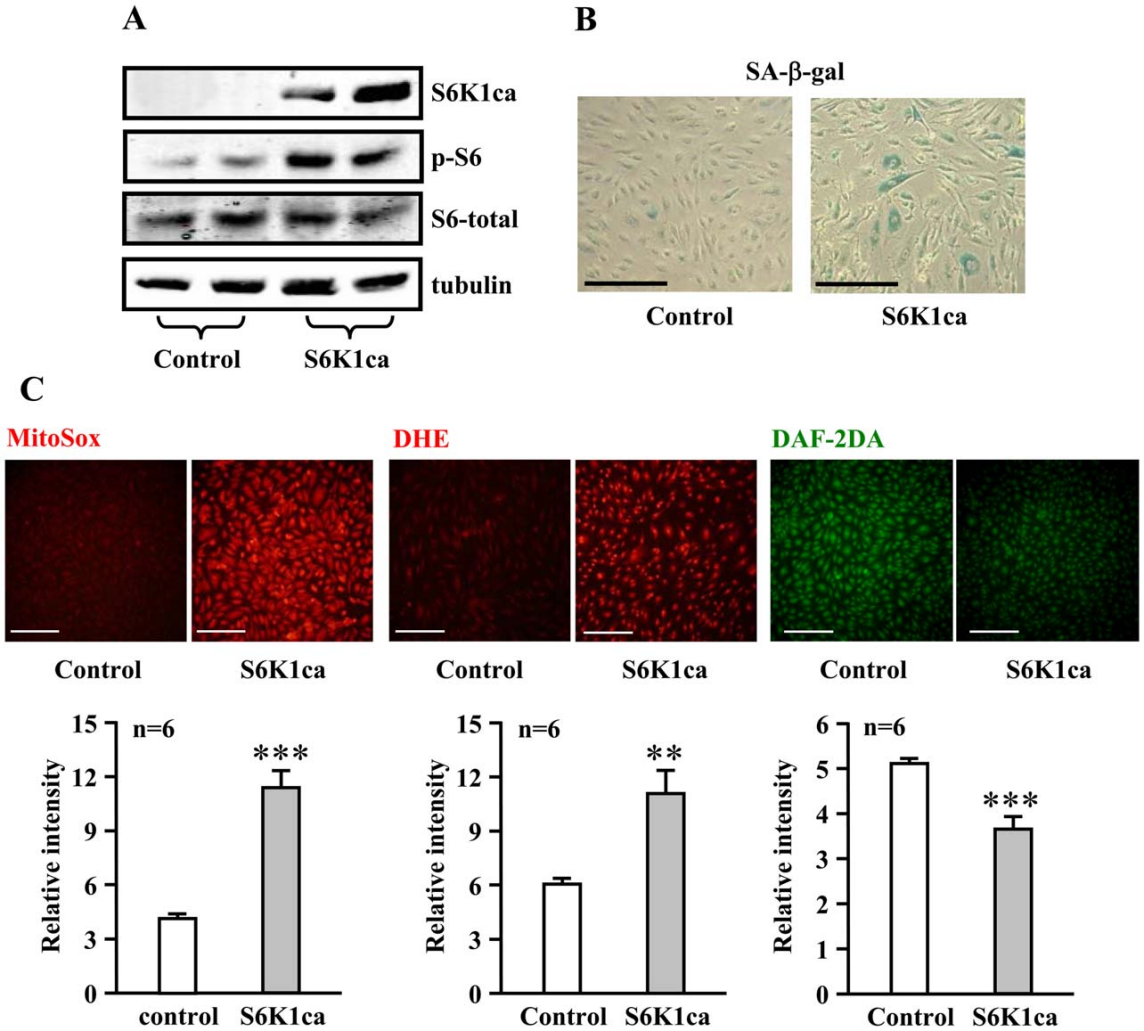
Over-expression of an active S6K1 mutant enhances superoxide, decreases NO production and induces premature senescence in young endothelial cells. Young HUVECs were transduced either with an empty rAd vector as control or with rAd/CMV-HA-S6K1-ca (a constitutively active S6K1 mutant). (**A**) Immunoblotting analysis of expression of HA-S6K1-ca with anti-HA antibody or antibodies against S6-S235/S236 (p-S6), total S6, and tubulin on day 2 of post transduction. (**B**) Four days post transduction, cells were subjected to SA-ß-gal staining. (**C**) MitoSox, DHE, and DAF-2DA staining were performed on day 2 of post transduction. Quantification of the signals is shown in the corresponding lower panels. n = 6. **p<0.01 and ***p<0.001 vs. control cells. Scale bar = 0.2 mm.

**Figure 6:**
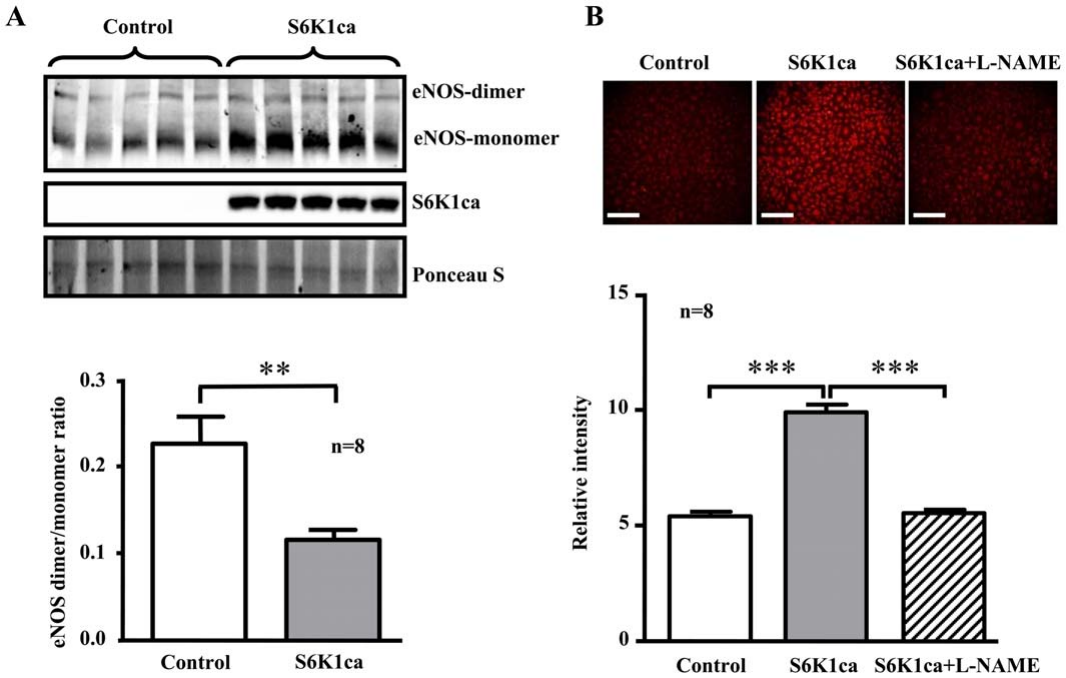
Over-expression of an active S6K1 mutant causes eNOS uncoupling in endothelial cells. Young HUVEC cells were transduced either with an empty rAd vector as control or with rAd/CMV-HA-S6K1ca (a constitutively active S6K1 mutant). Two days post transduction, cells were subjected to (**A**) Immunoblotting analysis of expression of eNOS-dimers, -monomers with anti-eNOS antibody, HA-S6K1-ca with anti-HA antibody. Ponseau S staining served as loading control. Quantification of the eNOS-dimer/monomer ratio is shown in the lower panel. n = 8 in each group. (**B**) DHE staining was performed 1 hour after L-NAME (1 mmol/L) treatment. Quantification of the signals is shown in the corresponding lower panels. n = 8. **p<0.01 and ***p<0.001 between indicated groups. Scale bar = 0.2 mm.

Conversely, adenovirus-mediated ectopic expression of a constitutively active S6K1 mutant (S6K1ca) in the young cells, as validated by increased S6K1ca protein level and enhanced S6-S235/S236 phosphorylation (**Fig. 5A**), induced premature cellular senescence as shown by increased number of SA-ß-gal positive cells (**Fig. 5B**). An enhanced superoxide anion generation and decreased NO production were also observed in cells overexpressing the active S6K1 mutant (**Fig. 5C**). Moreover, over-expression of the active S6K1ca mutant in the young endothelial cells also increased eNOS monomer level and decreased eNOS-dimer/monomer ratio (**Fig. 6A**, n = 8). The enhanced superoxide anion production in S6K1ca-overexpressing cells as assessed by DHE staining was inhibited by the eNOS inhibitor L-NAME (**Fig. 6B**, n = 8). The results demonstrate the causal role of S6K1 in eNOS uncoupling.

### Resveratrol inhibits Akt/S6K1 and improves endothelial function in senescent cells

**Figure 7:**
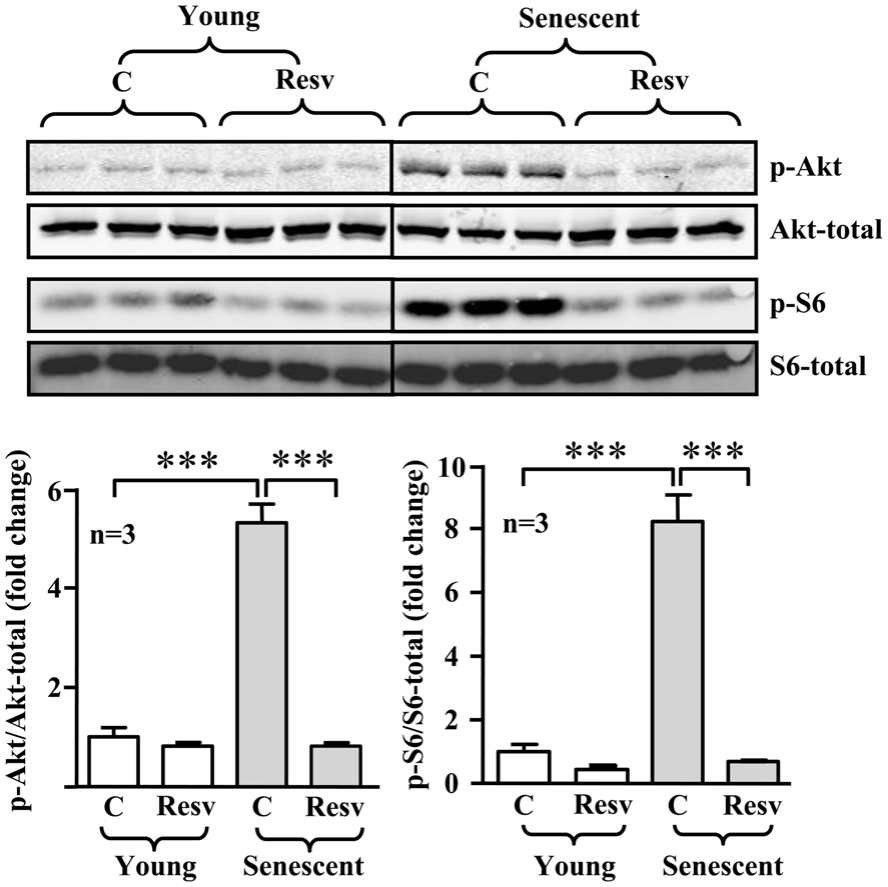
Resveratrol inhibits Akt and S6K1 signalling in senescent endothelial cells. Young or senescent HUVECs were treated with either solvent or resveratrol (Resv, 10 µmol/L) for one hour. The cell lysates were then prepared and subjected to immunoblotting analysis of Akt-S473 (p-Akt), total Akt, S6-S235/S236 (p-S6), and total S6. Quantification of the signals is shown in the lower panels. n = 6, ***p<0.001 between indicated groups.

**Figure 8:**
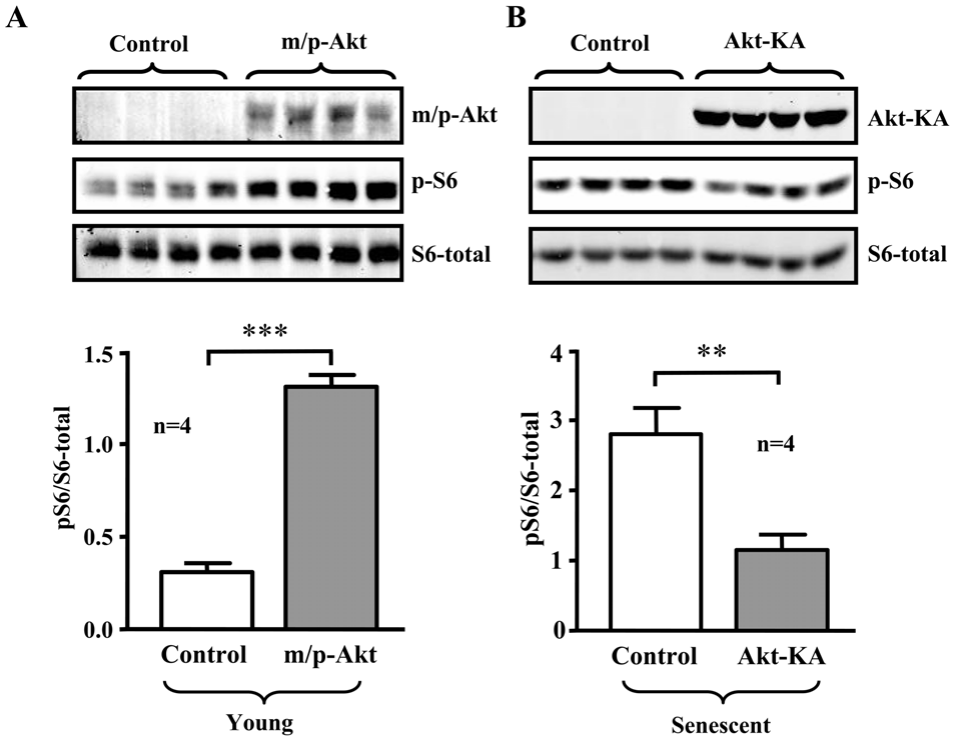
Role of Akt in S6K1 activation in endothelial cells. (**A**) Young HUVEC cells were transduced either with an empty rAd vector as control or with rAd/CMV-HA-m/p-Akt (a constitutively active Akt mutant). (**B**) Senescent HUVEC cells were transduced either with an empty rAd vector as control or with rAd/CMV-HA-Akt-KA (a dominant negative Akt mutant). Two days post transduction, the cells were subjected to immunoblotting analysis of expression of HA-tagged Akt mutants with anti-HA antibody, or with antibodies against S6-S235/S236 (p-S6) and total S6. Quantification of the signals is shown in the lower panels. n = 4. **p<0.01 and ***p<0.001 between indicated groups.

In senescent endothelial cells, the enhanced S6K1 activity was associated with an increased Akt activation, the upstream kinase of mTOR/S6K1, as measured by phosphorylation level of Akt-S473 (**Fig. 7**). The increased activation of both Akt and S6K1 in the senescent endothelial cells was inhibited by resveratrol (10 µmol/L, 1 hour) (**Fig. 7**). The role of Akt in S6K1 activation in endothelial cells was further demonstrated by experiments with HA-tagged Akt mutants. Over-expression of a constitutively active Akt mutant (m/p-Akt) in the young endothelial cells enhanced S6K1 activity as measured by increased level of p-S6-S235/S236 (**Fig. 8A**), whereas over-expression of a dominant negative mutant of Akt (Akt-KA) in the senescent endothelial cells decreased S6K1 activity (**Fig. 8B**).

**Figure 9:**
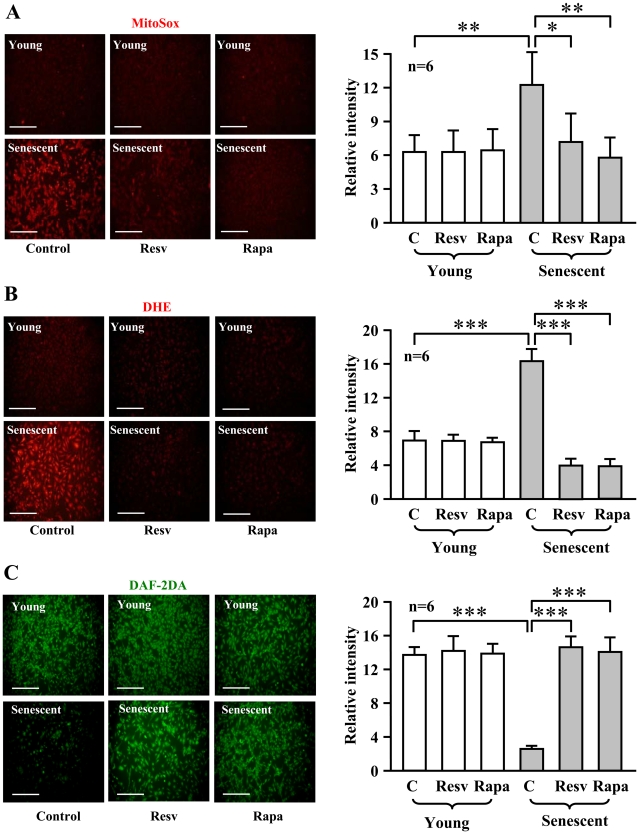
Resveratrol reduces superoxide generation and enhances NO production in senescent endothelial cells. Young and senescent HUVEC cells were treated with solvent as control (C), resveratrol (Resv, 10 µmol/L) or rapamycin (Rapa, 20 ng/ml) for one hour and then subjected to (**A**) MitoSox, (**B**) DHE, and (**C**) DAF-2DA staining. Quantification of the signals from six independent experiments is shown in the corresponding right panels. *p<0.05, **p<0.01 and ***p<0.001 between indicated groups. Scale bar = 0.2 mm.

Furthermore, the higher superoxide production in the senescent cells was blunted by resveratrol (10 µmol/L, 1 hour) as well as by rapamycin (20 ng/ml, 1 hour) (**Fig. 9A and 9B**), while decreased basal NO production in the senescent cells was restored by the two compounds (**Fig. 9C**). It is to note that resveratrol and rapamycin did not affect endothelial function in young cells (**Fig. 9**).

### Increased S6K1 activity in aging rat aortas: inhibition by resveratrol and rapamycin

**Figure 10:**
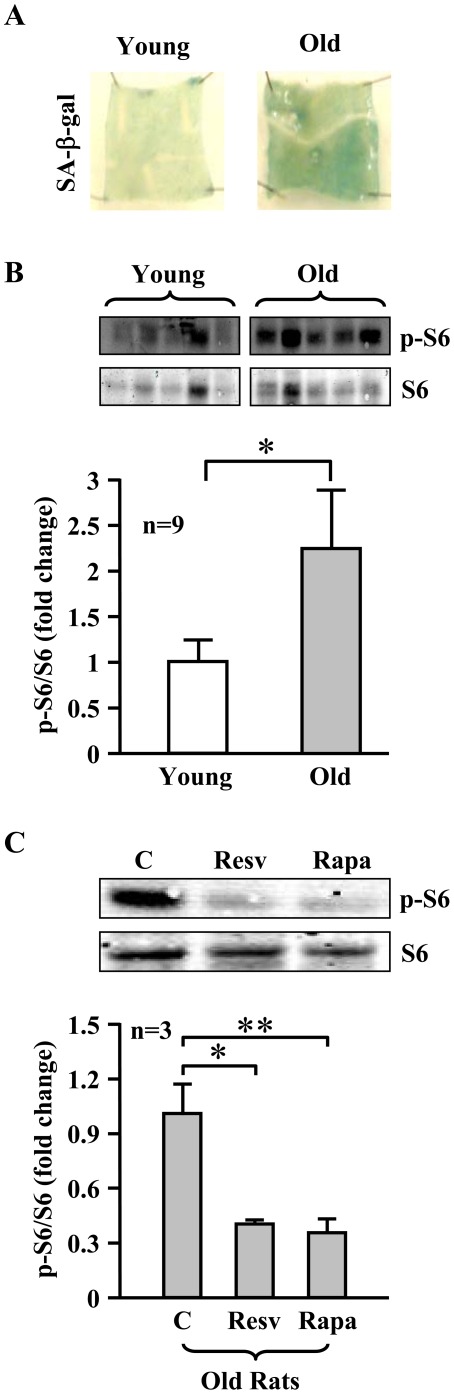
Increased S6K1 activity in aortas of old rats is inhibited by resveratrol. (**A**) SA-ß-gal staining of the endothelium in an aorta segment of a young and an old WKY rat. (**B**) Immunoblotting analysis of S6-S235/S236 (p-S6) and total S6 in the aortas of young and old rats (n = 9 of each group). (**C**) The aortas of old rats were treated *ex vivo* with solvent as control (C), resveratrol (Resv, 10 µmol/L) or rapamycin (Rapa, 20 ng/ml) in Krebs-Ringer bicarbonate solution (37°C, 95% O_2_/5% CO_2_) for one hour. The lysates were prepared for Immunoblotting analysis of S6-S235/S236 (p-S6) and total S6 levels (n = 3). *p<0.05 and **p<0.01 between indicated groups.

We then further investigated whether the above observations in cultured endothelial cells would also hold true in an aging animal model. The endothelium of old WKY rats (20–24 months old) had much stronger SA-ß-gal staining than that of young rats (1–3 months old) (**Fig. 10A**). As in the cell culture model, the S6K1 activity as assessed by measuring the level of S6-S235/S236 phosphorylation was significantly higher in the aortas of old WKY rats when compared to those of young rats (**Fig. 10B**). Moreover, the increased S6-S235/S236 phosphorylation level in the old rats was reduced by treatment of aortas with resveratrol (10 µmol/L, 1 hour) as well as with rapamycin (20 ng/ml, 1 hour) **(** **Fig. 10C**).

### Resveratrol and rapamycin inhibit oxidative stress and improve NO levels in aging rat aortas

**Figure 11:**
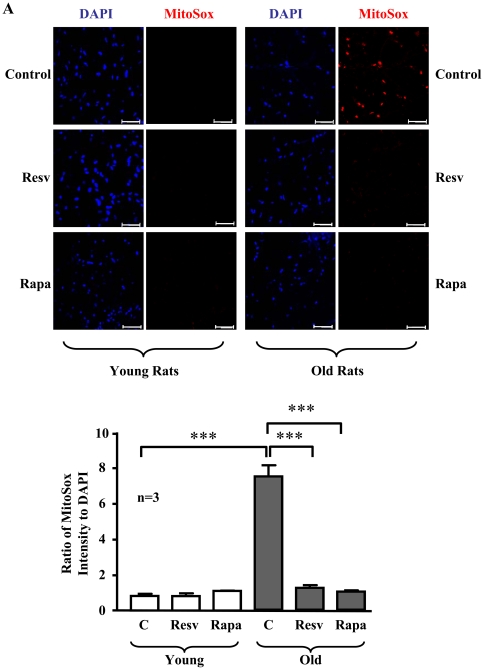
Inhibition of S6K1 suppresses superoxide generation in the aortas of old rats. Confocal microscopic *en face* detection of MitoSox staining for mitochondrial superoxide anion production followed by counterstaining with DAPI for endothelial nuclei in the intact thoracic aorta segments from young and old rats, which were treated with resveratrol (Resv) or rapamycin (Rapa) as described in Fig. 10. The upper panels show representative images from a young and an old rat and lower panel shows quantification of the fluorescence intensity from three animals in each group. Scale bar = 50 µm. ***p<0.001 between indicated groups.

**Figure 12:**
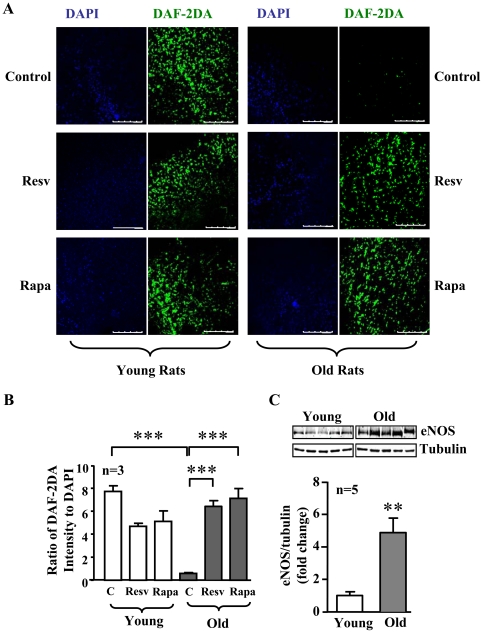
Inhibition of S6K1 increases NO production in the aortas of old rats. (**A**) Confocal microscopic *en face* detection of DAF-2DA staining for endothelial NO production followed by counterstaining with DAPI for endothelial nuclei in the intact thoracic aorta segments from young and old rats, which were treated with resveratrol (Resv) or rapamycin (Rapa) as described in Fig. 7. Shown are representative images from a young and an old rat. (**B**) Quantification of the fluorescence intensity from three animals in each group. Scale bar = 250 µm. ***p<0.001 between indicated groups. (**C**) Immunoblotting analysis of eNOS protein levels in aortas of young and old rats. Quantification of the signals is shown in the lower panel. n = 5, **p<0.01 vs. young rats.

**Figure 13:**
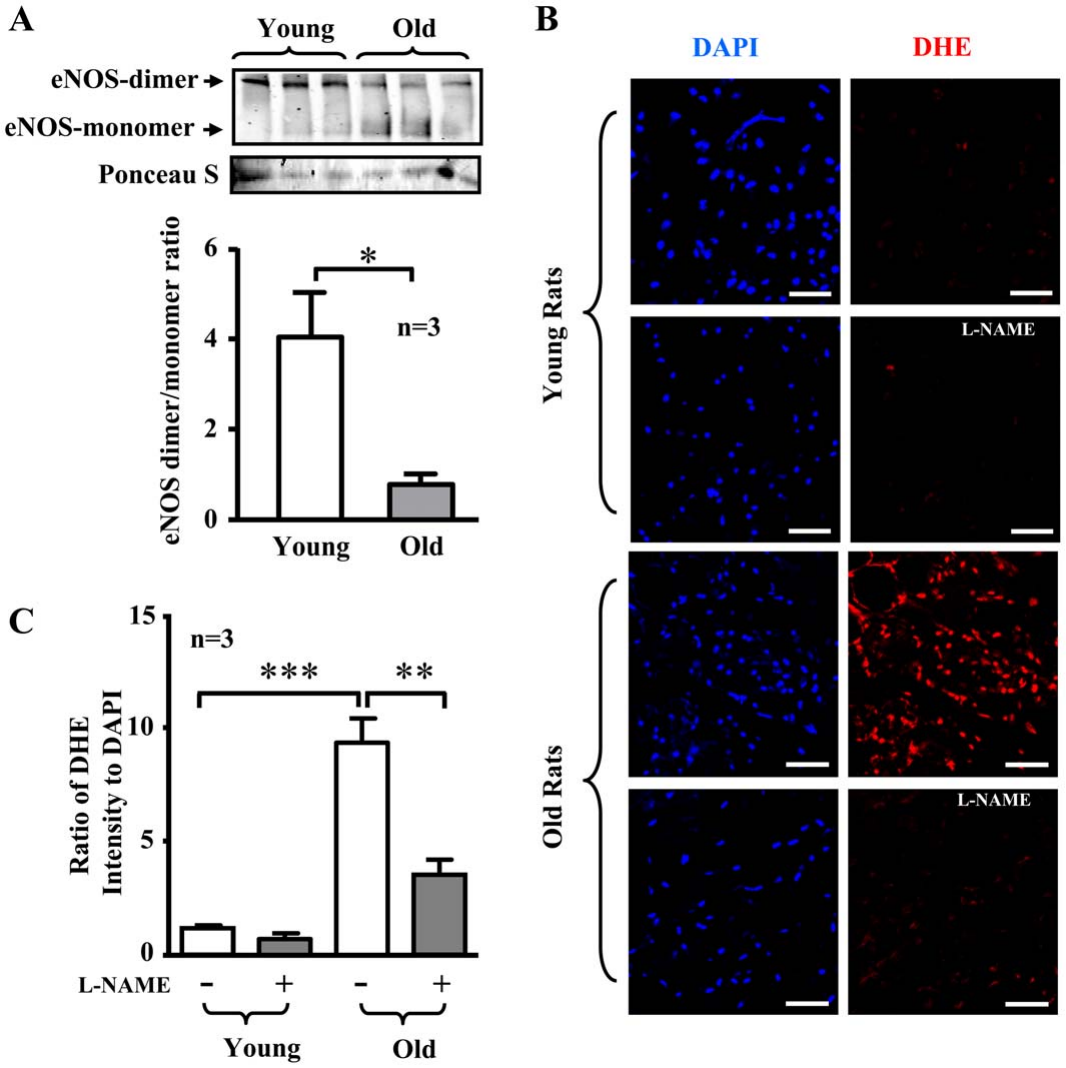
eNOS uncoupling in the aortas of old rats. (**A**) Immunoblotting analysis of eNOS-dimers and –monomers in the aortic endothelium of young and old rats. Ponseau S staining served as loading control. Quantification of the eNOS-dimer/monomer ratio is shown in the lower panel. n = 3 in each group. (**B**) Confocal microscopic *en face* detection of DHE staining for cytoplasmic superoxide anion production followed by counterstaining with DAPI for endothelial nuclei in the intact thoracic aorta segments from young and old rats, which were treated with or without L-NAME (1 mmol/L, 1 hour). Shown are representative images from a young and an old rat. (**C**) Quantification of the fluorescence intensity from three animals in each group. Scale bar = 50 µm. *p<0.05, **p<0.01, ***p<0.001 between indicated groups.

As compared to aortas of young rats, aortas of old rats displayed an increase in superoxide production as visualized by MitoSox staining, which was inhibited by treatment of the blood vessels with rapamycin or resveratrol (**Fig. 11**). Moreover, a decreased NO production detected by DAF-2DA staining in the aortas of old rats were improved by resveratrol or rapamycin (**Fig. 12A and 12B**), while no significant changes in NO production upon treatment were observed in the young rats (**Fig. 12A and 12B**). As observed in senescent endothelial cells, eNOS expression in aortas of old rats was also significantly increased as compared with that of young rats (**Fig. 12C**). Similar to the senescent endothelial cells in culture, a much higher eNOS monomer levels were observed in the aortic endothelium of old rats than that of the young rats (**Fig. 13A**). Conversely, eNOS dimers were decreased in the old rat aortic endothelium as compared to the young rats (**Fig. 13A**). Furthermore, as compared to the young rats, the endothelial production of superoxide anion in the aortas of old rats as assessed by *en face* DHE staining was significantly increased, which was largely reduced by treatment of the aortas with L-NAME (1 mmol/L, 1 hour, **Fig. 13B**).

**Figure 14:**
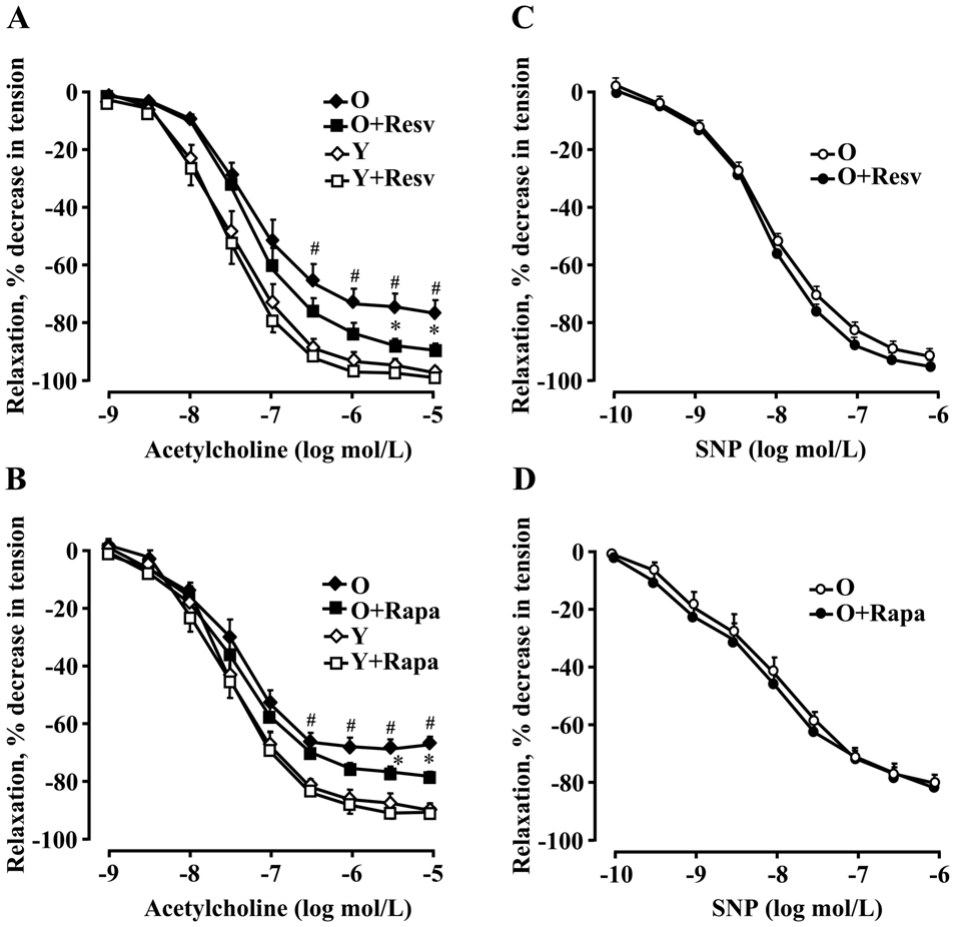
Inhibition of S6K1 signalling improves vascular endothelial function in old rats. (**A and B**) Endothelium-dependent relaxations to acetylcholine in aortas of old (O) and young (Y) rats (in the presence of indomethacin 1 µmol/L to eliminate endothelium-derived contracting prostanoids in aging) in the absence or presence of resveratrol (Resv, 10 µmol/L, for 1 hour) or rapamycin (Rapa, 20 ng/ml, for 1 hour), respectively. (**C and D**) Endothelium-independent relaxations in response to the NO-donor sodium nitroprusside (SNP) in aortas of old rats (O) in the absence or presence of either of the two drugs. n = 5 to 7; *p<0.05 between O and O+Resv or O+Rapa; # p<0.05 between old (O) and young (Y) rats.

Furthermore, the endothelium-dependent relaxations to acetylcholine (in the presence of 1 µmol/L indomethacin to inhibit aging-associated production of vasoconstrictor prostanoids which interferes with the effect of endothelium-derived NO [3] was impaired as compared to young animals (**Fig. 14A and 14B**). The endothelial function in old rats (not in young animals) was significantly improved by *ex vivo* treatment of aorta with resveratrol (10 µmol/L, 1 hour) or rapamycin (20 ng/ml, 1 hour) (**Fig. 14A and 14B**, respectively), whereas smooth muscle function as assessed by endothelium-independent relaxations to the NO-donor sodium nitroprusside (SNP) in old rats was not affected by either of the two compounds (**Fig. 14C and 14D**).

## Discussion

In the present study, we provide evidence showing that enhanced S6K1 signalling plays a causal role in endothelial aging and endothelial dysfunction related to eNOS uncoupling in a cell culture model and in a rat aging model. Furthermore, we demonstrate a novel mechanism of the vasoprotective effects of the polyphenol resveratrol in endothelial aging, namely improvement of endothelial function in aging through inhibition of S6K1.

Persistent activation of mTOR/S6K1 signalling has been shown to be associated with organism aging and aging-associated pathologies such as cancer, left ventricular hypertrophy, obesity, and muscle degeneration in animal models [5], [6], [13], [14]. Conversely, inhibition of mTOR or S6K1 extends lifespan and delays onset of aging-associated disorders in animal models [5], [6], [13], [14], [30]. Although the role of mTOR/S6K1 in regulation of lifespan of various organisms and animal models has been well documented, it is, however, not known whether mTOR, particularly S6K1, is involved in vascular endothelial aging and associated endothelial dysfunction. We show here that S6K1 activity is indeed enhanced in two models of vascular aging, namely in the senescent human endothelial cells in culture and in aortas of old rats. Moreover, we provide several lines of experimental evidence demonstrating that hyperactive S6K1 signalling plays a causal role in endothelial aging, oxidative stress, and decreased NO production: (a) Inhibition of S6K1 either by the mTOR inhibitor rapamycin or by S6K1 silencing in senescent endothelial cells reduced mitochondrial superoxide generation (evidenced by MitoSox staining) and enhanced NO production; (b) Treatment of aortas of old rats but not young rats with rapamycin resulted in inhibition of endothelial mitochondrial superoxide anion production and enhancement of basal NO production as well as improvement of endothelium-dependent relaxations to acetylcholine (stimulated NO production), demonstrating a critical role of S6K1 in endothelial oxidative stress and dysfunction in aging; (c) Over-expression of a constitutively active S6K1 mutant (S6K1ca) in young endothelial cells enhanced mitochondrial superoxide generation and decreased NO production, and induced premature senescence as demonstrated by increased number of SA-ß-gal positive cells in culture.

Increased superoxide production is well recognized as a mechanism for rapid inactivation of endothelial NO leading to endothelial dysfunction [31]. Multiple sources have been reported to contribute to enhanced superoxide production in endothelial cells including activated NADPH oxidase, mitochondrial dysfunction, and uncoupled eNOS [31]. With MitoSox Red indicator for specific detection of mitochondrial superoxide, we show here that S6K1 induces mitochondrial superoxide generation. This finding is in line with the role of mTOR in controlling mitochondrial ROS generation as reported in other cell types [32], [33]. The results of our present study demonstrate that mitochondrial ROS generation in aging is stimulated by persistent activation of S6K1. Moreover, the enhanced DHE signal in senescent endothelial cells suggests that hyperactive S6K1 is also capable of inducing superoxide generation from other sources.

In support of this notion, we show that S6K1 also enhances superoxide generation through eNOS uncoupling mechanism, since over-expression of the constitutively active S6K1 mutant in young endothelial cells leads to increased eNOS monomer level and decreased eNOS dimer/monomer ratio which are linked to eNOS uncoupling [34]. Most importantly, cytoplasmic superoxide generation as detected by DHE in the cells over-expressing S6K1 active mutant is markedly enhanced, which is abolished by the eNOS inhibitor L-NAME. Consistent with these results obtained from young endothelial cells over-expressing the active S6K1 mutant, in the senescent cells as well as in the aortas of old rats, in which S6K1 is hyperactive, an increased eNOS monomer level and decreased eNOS dimer/monomer ratio are observed. The associated increase in superoxide generation in the aging endothelial cells as assessed by DHE staining is largely inhibited by the eNOS inhibitor L-NAME. It is of note that eNOS protein level is higher in senescent endothelial cells and also in old rat aortas. Results on eNOS level in aging are controversial. Both increase and decrease in eNOS level in aging has been reported (see review article by Brandes et al. [2]). An increase in eNOS gene expression with dysfunctional enzymatic activity has also been demonstrated under several disease conditions such as atherosclerosis and diabetes [35]–[40]. The discrepancy on eNOS level in aging and the disease conditions is not clear and may depend on the stage of aging and the diseases. It seems that at the early or even advanced stage, eNOS level is not altered or even increased, the enzymatic activity of eNOS is, however, dysfunctional. At the very end of the disease stage, eNOS gene expression is decreased and contributes to the impairment of endothelial function [35], [38], [39]. The underlying mechanisms of increased eNOS level under the disease conditions and also in aging are not known. It has been hypothesized that oxidative stress under the physiological and pathological conditions may enhance eNOS level as a counteracting defense mechanism [38], [41], [42]. In the current study, we demonstrate that the higher eNOS level in aging endothelial cells is due to increased eNOS monomer levels which are enhanced by S6K1. The results together demonstrate that S6K1 plays a causal role in eNOS uncoupling in aging. Since activation of S6K1 leads to generation of superoxide from mitochondrion and from uncoupled eNOS, further experiments will be designed to investigate the relationship between mitochondrial dysfunction and eNOS uncoupling in aging.

It is well demonstrated that resveratrol exerts protective effects on aging-associated pathologies in animal models [20], [21] and possesses anti-oxidative properties in endothelial cells as shown in numerous previous studies including our own [43], [44]. In agreement with those studies, we show here that resveratrol inhibits mitochondrial superoxide generation and increases endothelial NO production in both aging models. Moreover, we demonstrate that resveratrol exerts similar inhibitory effects on S6K1 as rapamycin in the aortas of old rats. This is consistent with our data obtained in cultured senescent endothelial cells and with reports showing the inhibitory effect of resveratrol on S6K1 signalling in other cell types [24], [25], [27]. In view of the fact that hyperactive S6K1 is both necessary and sufficient for increased superoxide generation and decreased NO level in senescence/aging as evidenced in our current study, these data suggest that the beneficial effect of resveratrol on endothelial function in aging is at least in part attributable to its property of inhibiting S6K1 signalling. While the basal release of NO (as detected by DAF-2DA signal) can be fully restored by resveratrol and rapamycin, the stimulated release of NO in response to acetylcholine (as monitored in vascular reactivity study in a Multi-Myograph System) in old rat aortas is only partially improved by the two compounds. A probable explanation would be that additional defects in eNOS function upon activation with acetylcholine exist in aging. For example, co-factor i.e. BH4 deficiency seems to play an important role in aging-associated endothelial dysfunction [45].

Studies indicate that some pharmacological effects of resveratrol can be attributed to activation of the class III HDAC Sirt1 [22]. However, results from a recent study focusing on Sirt1 activity challenged the concept that Sirt1 is the direct target of resveratrol and related drugs [23]. In addition, increasing evidences have been presented that not all effects of resveratrol can be explained by stimulation of Sirt1. Sirt1-independent mechanisms have been reported [24], [25]. With respect to its inhibitory effect on mTOR/S6K1 signalling, both Sirt1-dependent and Sirt1-independent mechanisms have been demonstrated [46]. Evidences have been presented that inhibition of Akt, an upstream signalling of mTOR/S6K1 pathway, represents one of the mechanisms by which resveratrol negatively regulates mTOR/S6K1 pathway [47], [48]. Given that increased activity of Akt has been demonstrated to play a role in endothelial cell senescence [49], it is noteworthy that the increased S6K1 in senescence/aging in our current study is associated with increased basal Akt activity as evident by the increased phosphorylation of Akt. Moreover, resveratrol concomitantly inhibits Akt and S6K1 in the senescent endothelial cells in culture. These data suggest that the increased basal Akt activity accounts at least in part for the enhanced S6K1 activity in senescent/aging, and the inhibitory effect of resveratrol on S6K1 signalling may be exerted at least in part through inhibition of Akt. Indeed, over-expression of a constitutively active Akt mutant (m/p-Akt) in the young endothelial cells enhances S6K1 activity. Conversely, over-expression of a dominant negative mutant of Akt decreases, although not fully inhibits, S6K1 activity in the senescent endothelial cells. These results provide direct evidence for a role of Akt in activation of S6K1 in endothelial cells. An Akt-independent activation of S6K1 pathway in aging endothelial cells may also exist, which remains to be investigated in future studies.

In summary, our study demonstrates a causal role of S6K1 in eNOS uncoupling, endothelial oxidative stress, dysfunction, and cellular senescence in aging. The beneficial effects of resveratrol on vascular aging are at least partly mediated by inhibition of endothelial S6K1. Thus, inhibition of S6K1 signalling may represent a novel therapeutic approach for aging-associated vascular disease.

## Methods

### Ethics Statement

Animal work was approved by the Ethical Committee of Veterinary Office of Fribourg (number 174/07), Switzerland and was performed in compliance with guidelines on animal experimentation at our institution.

### Materials

All chemicals including those used for immunoblotting were obtained from Sigma (Buchs, Switzerland) except the following: l-norepinephrine bitartrate, acetylcholine (ACh), sodium nitroprusside (SNP), *trans*-resveratrol were purchased from Calbiochem; antibodies against phospho-S6K1-T389 (9205), phospho-S6-S235/236 (2211s), S6 (2217s) were from Cell Signalling (Allschwil, Switzerland); Antibodies against S6K1 (9205s) was from BD Transduction laboratories (Allschwil, Switzerland); Alexa Fluor680-conjugated anti-mouse IgG (A21057), dihydroethidium (DHE), MitoSox red mitochondrial superoxide indicator (MitoSox) were from Molecular Probes/Invitrogen (Lucerne, Switzerland), and IRDye800-conjugated anti-rabbit IgG (926–32211) were from LI-COR Biosciences (Bad Homburg, Germany); the membrane-permeable 4,5-diaminofluoresceine acetate (DAF-2DA) was from VWR international SA (Dietikon, Switzerland); x-gal was from Promega; Endothelial cell growth supplement (ECGS) pack was from PromoCell GmbH (Allschwil, Switzerland) and all cell culture media and materials were purchased from Gibco BRL (Lucerne, Switzerland).

### Animals

Young (1 to 3 months old) and old (20 to 24 months) male WKY rats (Harlan Netherlands) were maintained on a 12-h light-dark cycle and fed standard chow diet and tap water according to the local guidelines of animal experimentation. The animals were anesthetized with xylazin (10 mg/kg body weight, intraperitoneally) and ketamin (100 mg/kg body weight, intraperitoneally) and sacrificed. Thoracic aortas were dissected and cleaned from perivascular fat, and subjected to organ chamber experiments, *en face* staining or snap frozen directly in liquid N_2_ and kept at −80°C till further biochemical analyses.

### Recombinant adenoviral (rAd) expressing short hairpin RNA (shRNA), S6K1 or Akt

rAd expressing shRNA targeting human S6K1 driven by the U6 promoter (rAd/U6-S6K1^shRNA^) and rAd/CMV-HA-S6K1ca (a constitutively active S6K1 mutant F5A-E389-R3A), -HA-m/p-Akt (a constitutively active Akt mutant) and –HA-Akt-KA (a dominant negative Akt mutant) were described previously [17], [50], [51]. The control rAd expressing LacZ^shRNA^ and the empty rAd were from Invitrogen life Technologies.

### Senescence-associated ß-galactosidase (SA-ß-gal) staining

Cells were initially washed twice with PBS followed by fixation with 2% formaldehyde solution in PBS for 10–15 min. After washing twice with PBS, cells were then incubated with the SA-ß-gal staining solution (1 mg/ml X-gal, 40 mmol/L citric acid, 5 mmol/L potassium ferrocyanide, 5 mmol/L potassium ferricyanide, 150 mmol/L sodium chloride, 2 mmol/L magnesium chloride dissolved in phosphate buffer, pH 6.0) overnight at 37°C in a CO_2_-free atmosphere. The stained senescent cells were detected by conventional microscopy.

### Endothelial cell culture and adenoviral transduction of the cells

Human umbilical vein endothelial cells (HUVEC) [36] were maintained in RPMI-1640 medium supplemented with 5% FCS and ECGS. Cells of passage 1 to 2 (P1 to P2) were used as young cells. Some of the cells were further splitted in a ratio of 1∶3 continuously over a period of several weeks till replicative senescence was reached as assessed by SA-ß-gal staining (P9 to P12). Transduction of HUVECs by recombinant adenovirus was performed as previously described [51]. Cells were transduced with the recombinant adenovirus at titers of 100 Multiplicity Of Infection (MOI) and cultured in complete medium for 2–4 days before experiments as indicated in the figures.

### Immunoblotting

Cell or tissue lysate preparation, SDS-PAGE, transfer of SDS gels to an Immobilon-P membranes (Millipore) were performed as previously described [36]. The resultant membrane was first incubated with the corresponding primary antibody at room temperature for 2 hours with gentle agitation after blocking with 5% skimmed milk. The blot was then further incubated with a corresponding anti-mouse (Alexa fluor 680 conjugated) or anti-rabbit (IRDye 800 conjugated) secondary antibodies. Signals were visualized using Odyssey Infrared Imaging System (LI-COR Biosciences). Quantification of the signals was performed using NIH Image 1.62 software.

### Detection of eNOS-monomer and dimer in cultured endothelial cells and the endothelium from rat aortas

Low-temperature SDS-PAGE (LT-PAGE) was performed for detection of SDS-resistant eNOS dimer and monomer as described previously [52]. Briefly, cultured endothelial cells or the endothelial layers isolated from rat thoracic aortas treated with collagenase (collagenase type IA, 1 mg/ml in RPMI for 20 min) were harvested in the cold extraction buffer as described above for immunoblotting. Equal amount of protein of each sample was treated with 1xLaemmli buffer without 2-mercaptoethanol or DTT on ice and subjected to 6%SDS-PAGE at 4°C in a cold room. Subsequent to LT-PAGE, immnunoblotting was performed with anti-eNOS antibody as described above.

### Activity of S6K1

Activity of S6K1 was analyzed by monitoring phosphorylation of its substrate S6 at serine235/serine236 (S235/S236) by immunoblotting.

### Detection of NO and superoxide level in cultured endothelial cells

For detection of NO, HUVECs were gently washed twice with Ca^2+^-free PBS, and incubated in a modified Krebs-Ringer bicarbonate solution (in mmol/L, NaCl 118, KCl 4.7, CaCl_2_ 2.5, MgSO_4_ 1.2, KH_2_PO_4_ 1.2, NaHCO_3_ 25, EDTA 0.026, and glucose 5.5) containing 5 µmol/L of DAF-2DA for 30 minutes. For measurement of superoxide, cells were incubated with 5 µmol/L DHE for 20 minutes or 5 µmol/L MitoSox for 10 minutes for detection of cytoplasmic or mitochondrial superoxide, respectively. For functional eNOS uncoupling study, cells were pre-treated with the eNOS inhibitor L-NAME (1 mmol/L) for 1 hour before DHE was added. The cells were then washed 3 times and images were obtained with Zeiss fluorescence microscopy. The intensity of the fluorescence was quantified by Image J software (U. S. National Institutes of Health).

### *en face* Detection of NO and superoxide in rat aortas

NO and mitochondrial superoxide production in the absence or presence of resveratrol or rapamycin was assessed with DAF-2DA and MitoSox staining, respectively. Briefly, young (1–3 months) and old rat (20–24 months) aortas cleaned of perivascular tissues were equilibrated for 30 minutes in Krebs buffer at 37°C aerated with 95% O_2_ and 5% CO_2_. After equilibration, resveratrol (10 µmol/L) and rapamycin (20 ng/ml) were added to respective blood vessels for 1 hour. DAF-2DA (5 µmol/L) or MitoSox (5 µmol/L) was then added for 30 minutes or 10 minutes, respectively. For functional eNOS uncoupling study, aortas of young and old rats were treated as above described, except that L-NAME (1 mmol/L) was added to respective blood vessels for 1 hour followed by staining with DHE (5 µmol/L) for 20 minutes. The aortas were then washed three times and fixed in 4% paraformaldehyde followed by counterstaining with DAPI (300 nmol/L for 3 minutes). After washing with Krebs buffer, the aortas were carefully cut longitudinally and mounted *en face* (face down) on slides and then covered with cover slip for endothelial layer imaging. Vectashield mounting medium was used to preserve the fluorescence. The fluorescence was analyzed in a Leica DM6000 confocal microscope within hours after preparation. Fluorescence from DAF-2DA staining was excited with 488 nm argon laser with emission detection at 500–535 nm, whereas fluorescence from MitoSox was detected at 590 nm emission. Z-scanning was done for each sample. After the signal on the top (endothelial layer on the lumen border) of the sample was observed, the images were collected. Three consecutive images per field, acquired through the full thickness of endothelial signal, were recorded for analysis. At least 3 different fields per sample were evaluated. The images from DAF-2DA, MitoSox and DAPI staining were quantified with Image J software and results are presented as the ratio of DAF-2DA and DAPI positive nucleus or ratio of MitoSox and DAPI.

### Endothelium-dependent and -independent relaxations

Aortas were isolated from young and old rats and cleaned of perivascular tissues as described [43]. Briefly, the aortas with endothelium were cut into rings (3 mm in length) and suspended in a modified Krebs-Ringer bicarbonate solution aerated with 95% O_2_ and 5% CO_2_ at 37°C in a Multi-Myograph System (Model 610 M, Danish Myo Technology A/S, Denmark) in parallel. The aortic rings were allowed to equilibrate for 45 minutes and progressively stretched to a passive tension of 10 mN that gives the optimal length–tension relationship. To study the effects of resveratrol or rapamycin on endothelium-dependent or independent relaxations, one ring was incubated with vehicle as control, the others were treated with either resveratrol (10 µmol/L) or rapamycin (20 nmol/L) for 1 hour. The aortic rings were then contracted with norepinephrine (0.3 µmol/L). After the contraction induced by norepinephrine reached plateau, cumulative concentration-responses to acetylcholine (ACh; 1 nmol/L to 10 µmol/L) or to the NO donor sodium nitroprusside (SNP; 0.1 nmol/L to 10 µmol/L) were performed in each aortic ring. Indomethacin (1 µmol/L) was added to the aortic rings throughout the whole experiments to prevent any generation of endothelium-derived contracting prostanoids.

### Statistics

Data are given as mean±SEM. In all experiments, n represents the number of experiments or animals. Statistical analysis was performed with unpaired t test or ANOVA with Dunnett or Bonferroni post-test. For analysis of vascular relaxations to ACh in isolated aortas, relaxation responses to the same concentration of ACh among the different aortic rings were compared. Statistical analysis was performed using ANOVA with Bonferroni post-test. Differences in mean values were considered significant at p<0.05.
